# A Novel Small Molecule Inhibitor of Hepatitis C Virus Entry

**DOI:** 10.1371/journal.ppat.1001086

**Published:** 2010-09-02

**Authors:** Carl J. Baldick, Michael J. Wichroski, Annapurna Pendri, Ann W. Walsh, Jie Fang, Charles E. Mazzucco, Kevin A. Pokornowski, Ronald E. Rose, Betsy J. Eggers, Mayla Hsu, Weixu Zhai, Guangzhi Zhai, Samuel W. Gerritz, Michael A. Poss, Nicholas A. Meanwell, Mark I. Cockett, Daniel J. Tenney

**Affiliations:** Bristol-Myers Squibb, Research and Development, Wallingford, Connecticut, United States of America; Washington University School of Medicine, United States of America

## Abstract

Small molecule inhibitors of hepatitis C virus (HCV) are being developed to complement or replace treatments with pegylated interferons and ribavirin, which have poor response rates and significant side effects. Resistance to these inhibitors emerges rapidly in the clinic, suggesting that successful therapy will involve combination therapy with multiple inhibitors of different targets. The entry process of HCV into hepatocytes represents another series of potential targets for therapeutic intervention, involving viral structural proteins that have not been extensively explored due to experimental limitations. To discover HCV entry inhibitors, we utilized HCV pseudoparticles (HCVpp) incorporating E1-E2 envelope proteins from a genotype 1b clinical isolate. Screening of a small molecule library identified a potent HCV-specific triazine inhibitor, EI-1. A series of HCVpp with E1-E2 sequences from various HCV isolates was used to show activity against all genotype 1a and 1b HCVpp tested, with median EC_50_ values of 0.134 and 0.027 µM, respectively. Time-of-addition experiments demonstrated a block in HCVpp entry, downstream of initial attachment to the cell surface, and prior to or concomitant with bafilomycin inhibition of endosomal acidification. EI-1 was equally active against cell-culture adapted HCV (HCVcc), blocking both cell-free entry and cell-to-cell transmission of virus. HCVcc with high-level resistance to EI-1 was selected by sequential passage in the presence of inhibitor, and resistance was shown to be conferred by changes to residue 719 in the carboxy-terminal transmembrane anchor region of E2, implicating this envelope protein in EI-1 susceptibility. Combinations of EI-1 with interferon, or inhibitors of NS3 or NS5A, resulted in additive to synergistic activity. These results suggest that inhibitors of HCV entry could be added to replication inhibitors and interferons already in development.

## Author Summary

Approximately 170 million people worldwide are chronically infected with hepatitis C virus (HCV), which is a leading cause of chronic liver disease. Current treatments are not optimal; however, several molecules that inhibit HCV replication are in development. However, resistance to individual antivirals is likely to develop, requiring therapy consisting of a combination of drugs targeting different stages of the viral life cycle. The entry of HCV into hepatocytes is a multistep process, involving at least four cellular receptors, leading to virion endocytosis and fusion of the viral and cellular membranes. Unlike the HCV replication process, these steps have not been thoroughly exploited as targets for antiviral intervention. Therefore, we screened a small molecule library for inhibitors of HCV entry and identified a compound, EI-1, that potently blocked genotype 1a and 1b HCV infection. Importantly, EI-1 also prevented direct cell-to-cell spread of HCV, a potentially significant route of transmission in infected livers. In addition, our studies suggest that EI-1 susceptibility is mediated by the viral E2 envelope glycoprotein, as resistance in E2 can overcome inhibition. The antiviral activity of EI-1 is potentiated by combinations with other HCV inhibitors, demonstrating the value of entry inhibitors in potential combination antiviral regimens.

## Introduction

Hepatitis C virus (HCV), a member of the *Flaviviridae* family of positive-strand RNA viruses, chronically infects approximately 170 million people worldwide [1], [2]. Over time, ongoing virus replication within the liver often leads to severe clinical manifestations such as fibrosis, cirrhosis, and hepatocellular carcinoma [3], [4]. Consequently, HCV-induced disease is the leading indication for liver transplantation [5]. Medical treatment for HCV is limited by the lack of a vaccine or approved therapies that specifically target the virus. Currently, patients undergo treatment with a combination of parenterally administered pegylated interferon-alpha (IFN-α) and oral ribavirin [6]. Genotype 1 HCV has proven to be the most difficult to treat and elimination of the virus (sustained virologic response) is achieved for only approximately 50% of patients [7], [8]. This poor treatment response, combined with often severe side effects induced by therapy, highlight a need for improved antiviral drugs with better efficacy and safety profiles.

Studies with isolated HCV replication enzymes and replicon cell-based systems have been exploited to identify several inhibitors of HCV replication that are currently in clinical development [9]. While these have demonstrated potent reduction of circulating virus in early clinical trials, preexisting or rapidly-emerging resistance is a characteristic of the highly mutable HCV genome [9], [10]. As with HIV treatment paradigms, these results dictate that combination therapy, targeting multiple stages or functions of the HCV infection cycle, will be required to treat HCV. Therefore, we sought to search for inhibitors that could complement those currently in development.

HCV encodes two envelope glycoproteins, E1 and E2, which together mediate binding and entry of the virus into primary hepatocytes and hepatocyte cell lines. The sequence of events leading to virus internalization has not been completely defined, but recent evidence implicates several cell surface molecules in the process. The initial attachment (adsorption) of the virus is likely facilitated by a low affinity interaction of E2 with heparan sulfate proteoglycans (HSPGs) on the cell surface [11], [12], [13]. Subsequently, higher affinity interactions with several host cell surface receptors have been shown to be required for HCV entry. These include the low-density lipoprotein receptor (LDL-R) [14], [15], [16], the tetraspanin CD81 [17], [18], [19], [20], [21], [22], scavenger receptor class B type I (SR-BI) [18], [19], [20], [23], [24], [25], [26], and tight junction proteins claudin (CLDN)-1, 6, or 9, and occludin [27], [28], [29], [30]. *In vitro*, non-permissive human, mouse, and hamster cell lines become permissive to infection with HCV pseudoparticles (HCVpp) if engineered for ectopic expression of SR-BI, CD81, CLDN1, and occludin suggesting these are the core HCV receptors required for entry of the virus into hepatocytes [29]. The LDL-R is postulated to function primarily in the context of serum HCV particles, and may facilitate the attachment or uptake of virions which are complexed with very low density lipoproteins *in vivo* [31]. There is some evidence to suggest that HCV interacts with CD81 and SR-BI earlier in the entry pathway, followed by CLDN1 and occludin at tight junctions, although the exact order of binding and the role of each receptor remain to be determined [18], [23], [27].

Following receptor binding, virions are internalized *via* clathrin-mediated endocytosis [32], [33], [34], [35]. By analogy to the phylogeneticlly related flaviviruses [36], [37], [38], the reduced pH of the endosome is thought to mediate a conformational change in the HCV virion that facilitates fusion of the viral and endosome membranes, depositing the nucleocapsid into the cytoplasm. Indeed, agents that prevent the acidification of the endosome block HCV entry if added within 3 hr after infection [39]. Structural features characteristic of class II viral fusion glycoproteins of flaviviruses and alphaviruses [40], [41] have been identified within HCV E1 and E2, and it remains to be determined if one or both of these proteins mediate the fusion process [42], [43], [44], [45], [46].

HCVpp, which consist of retroviral or lentiviral cores surrounded by an envelope containing HCV E1 and E2, have proven to be a valuable surrogate system by which to study the viral and cellular determinants of the viral entry pathway [47], [48]. The early steps of infection by infectious cell culture HCV (HCVcc), including receptor binding, internalization, and pH-dependent endosomal fusion, are mimicked by HCVpp. In addition, pseudoparticles can be engineered to express reporter proteins, affording a convenient system to quantify E1E2-mediated entry in the absence of other HCV-encoded functions. The HCVpp system is easily amenable to genetic manipulation of E1 and E2, allowing the characterization of envelope protein genotype variation and identification of functionally important regions through mutagenesis.

The molecular targets for current HCV direct-acting antiviral agents in drug development are focused on the non-structural proteins required for replication such as the NS3 protease, NS5A, and the NS5B RNA-dependent RNA polymerase. The viral entry pathway encompasses several additional potential points for intervention, and therapies targeting entry would provide a differentiated mechanism that could be a component of future drug combination regimes [49]. Here, we characterize a small molecule entry inhibitor identified through a high-throughput HCVpp screening effort. The inhibitor is most potent against genotype 1 HCV and functions at a post-HSPG binding step, prior to or concomitant with fusion. Using chimeric HCVcc expressing genotype 1a, 1b, or 2a envelope proteins, we demonstrate comparable potency and the ability to block cell-to-cell spread. HCVcc resistant to this molecule was isolated and amino acid changes were mapped to a residue in the transmembrane domain (TMD) of E2, distinct from regions identified in receptor-binding functions. Combination of the entry inhibitor with IFN-α or other HCV-specific antivirals resulted in additive to synergistic activity.

## Materials and Methods

### Cells and culture conditions

293T cells (ATCC, Manassas, VA), Huh-7 cells (a gift from Ralf Bartenschlager), and Huh-H1 cells were maintained in Dulbecco's Modified Eagle Medium (DMEM) (Invitrogen, Carlsbad, CA) supplemented with 10% fetal bovine serum (FBS) (HyClone, Logan, UT), 2 mM L-glutamine, 1mM sodium pyruvate, 10% nonessential amino acids, 10 mM HEPES, 100 units/ml penicillin, and 100 units/ml streptomycin. Huh-7.5 cells (Apath, Brooklyn, NY) were maintained in DMEM containing 10% FBS, 10% nonessential amino acids, 100 units/ml penicillin, and 100 units/ml streptomycin. Madin-Darby canine kidney (MDCK) cells (ATCC) were maintained in Minimum Essential Medium (MEM) (Invitrogen, Carlsbad, CA) containing 5% FBS and 0.1% sodium bicarbonate. Madin-Darby bovine kidney (MDBK) cells (ATCC) were maintained in DMEM supplemented with 10% Ultra-Low IgG FBS (Invitrogen), 2 mM L-glutamine, 100 units/ml penicillin, and 100 units/ml streptomycin. MT2 cells were obtained from the NIH Research and Reference Reagent Program and maintained in RPMI 1640 supplemented with 10% FBS, 10 mM HEPES, 2 mM L-glutamine, 100 units/ml penicillin, and 100 units/ml streptomycin.

### Generation of cells expressing high levels of CD81

CD81 cDNA was isolated from Huh-7 cells by reverse transcriptase PCR (RT-PCR) and cloned under the transcriptional control of the CMV IE promoter in pcDNA3.1(-) (Invitrogen) to create p131-C1. Huh7B cells were transfected with p131-C1 using Lipofectamine 2000 (Invitrogen) according to the manufacturer's protocol and subjected to selection with 1 mg/ml G418. Limiting dilution and flow cytometry using anti-CD81 monoclonal antibody (BD Biosciences, San Jose, CA) were used to segregate the cells expressing the highest levels of CD81. A cell clone, designated here as Huh-H1, was expanded and maintained in media containing 0.5 mg/ml G418.

### Cloning of the HCV E1E2 coding sequences

HCV RNA was isolated from infected human sera, obtained following informed consent, using the QiaAmp Viral RNA Extraction kit (Qiagen, Valencia, CA) according to the manufacturer's instructions. HCV RNA was reverse transcribed using Thermoscript (Invitrogen) and genotype-specific primers designed using publically available sequences. Subgenomic amplicons harboring complete core-p7 regions were amplified using genotype-specific forward primers within the HCV 5′ UTR and reverse primers within NS2 or NS3. Sequences encoding the last 21 amino acids of core (E1 signal sequence) through the end of E2 were amplified using patient-specific primers and cloned into pcDNA3.1(+) (Invitrogen) for HCVpp production. HCVpp pseudotyped with envelope proteins from genotype 1b isolate 432-4 (GenBank accession number HM049503) were used for the compound library screening, as well as other experiments presented here. Prototypical laboratory HCVpp envelopes were derived from genotype 1a H77C (GenBank accession number AF009606), genotype 1b Con1 (GenBank accession number AJ238799), genotype 2a JHF1 (GenBank accession number AB047639), or genotype 2a J6 (GenBank accession number AF177036) and cloned into expression vector pcDNA3.1(+).

### Preparation of viral pseudoparticles

Murine leukemia virus (MLV)-based pseudoparticles containing HCV E1E2 or vesicular stomatitis virus (VSV) glycoprotein G envelope proteins were produced by a modification of published procedures [47], [48]. Briefly, 3.5×10^7^ 293T cells were transfected with 17.5 µg of pVPack-GP (Stratagene, La Jolla, CA) expressing the murine leukemia virus capsid and polymerase proteins, 17.5 µg of pFB-luc2 (a derivative of pFB-luc (Stratagene) in which the firefly luciferase gene was replaced with a human codon optimized firefly luciferase from pGL4.10 (Promega, Madison WI)) encoding an MLV genome expressing luciferase reporter gene, and 4.4 µg of either pVSV-G (Clontech, Mountain View, CA) expressing the VSV envelope glycoprotein G or one of the HCV E1E2-expressing plasmids (above). Transfections were performed for 6 hrs using Lipofectamine 2000 as described by the manufacturer (Invitrogen), at which time the medium was removed and replaced with DMEM/10% FBS. Media containing HCV pseudoparticles (HCVpp) or VSV pseudoparticles (VSVpp) was collected 3 days following transfection, clarified by passage through a 0.45 µm filter (Millipore, Bedford, MA), and stored at −70°C as a viral stock.

### HCVpp infection assays

For compound library screening, infections were performed in 384-well plates by mixing HCVpp or VSVpp with 1×10^4^ Huh-H1 cells/well in the presence or absence of test inhibitors, followed by incubation at 37°C. Luciferase activity, reflecting the degree of entry of the pseudoparticles into host cells, was measured 2 days after infection using the Steady-Glo Reagent (Promega). Test compounds were serially diluted 3-fold in dimethyl sulfoxide (DMSO) to give a final concentration range in the assay of 50.0 µM to 0.04 pM. Maximum activity (100% of control) and background were derived from control wells containing DMSO alone or from uninfected wells, respectively. The individual signals in each of the compound test wells were then divided by the averaged control values (wells lacking inhibitor), after background subtraction, and multiplied by 100% to determine percent activity. The corresponding % inhibition values were then calculated by subtracting this value from 100. Assays were performed in triplicate and average EC_50_ values (reflecting the concentration at which 50% inhibition of virus replication was achieved) were calculated using XLfit for Excel (ID Business Solutions, Burlington, MA). The specificity of the compounds for inhibiting HCV was determined by evaluating inhibition of VSVpp infection in parallel.

### Preparation of cell culture adapted HCV (HCVcc)

HCVcc utilized consisted of chimeric viruses containing structural genes from genotype 1a, 1b, or 2a and nonstructural regions from genotype 2a JFH-1. The full length JFH1 genome was chemically synthesized. The genotype 1a chimeric JFH1 virus containing the H77C structural region (HCV-1a/2a) was constructed as described [50]. The genotype 1b chimeric JFH1 virus, containing the core to the NS2 C3 junction from the genotype 1b isolate 432-4 (HCV-1b/2a), was constructed as described [51]. The genotype 2a chimeric JHF1 virus containing the J6CF structural region (HCV-2a/2a) was constructed as described [51]. Chimeric reporter viruses contained an in-frame *Renilla* luciferase gene (HCVcc-1a/2a-Rluc, HCVcc-1b/2a-Rluc, and HCVcc-2a/2a-Rluc) inserted in-between the NS5A and NS5B coding sequences such that the NS3 protease cleavage sequences were reconstituted on either side of luciferase (Burt Rose, unpublished). *In vitro* RNA was prepared from these cloned sequences using the MEGAscript kit (Ambion, Austin, TX) and transfected by electroporation into Huh-7.5 cells as described [52]. Media containing virus was collected, clarified by low speed centrifugation, and stored at −70°C. HCVcc titers were determined by infection of Huh-7.5 cells with serial dilutions of virus, followed by indirect immunofluorescence for HCV core protein as described below, and expressed as focus forming units (ffu)/ml. Virus preparations required several passages for adaptation required to generate high titer stocks.

### HCVcc infection assays

Infections utilizing HCVcc chimeras expressing the *Renilla* luciferase protein were quantified by infecting Huh-7.5 cells (with or without inhibitors), incubating at 37°C for 3 days, and measuring luciferase activity using the EnduRen substrate (Promega) as described by the manufacturer. Infections utilizing HCVcc without a reporter were quantified by indirect immunofluorescence. HCVcc was added to Huh-7.5 cells (with or without inhibitors) in special-optics, collagen-coated 96-well plates (BD Biosciences) and incubated at 37°C for 2–4 days. Cells were then washed twice with PBS and fixed with 4% paraformaldehyde in PBS (Sigma) for 30 min at room temperature. Following 2 washes in PBS, cells were permeabilized with 0.25% Triton X-100 (Pierce, Rockford, IL) in PBS for 10 min and blocked with 2% bovine serum albumen (Sigma) in PBS for 30 min. Samples were incubated for 2 hr with 3 µg/ml anti-HCV Core monoclonal antibody (ABR-Affinity Bioreagents, Golden, CO), washed 4 times with PBS, and incubated with a 1/500 dilution of Alexa Fluor 488-labeled donkey anti-mouse secondary antibody (Invitrogen) for 1 hr. Samples were washed three times with PBS and 0.5 µg/ml of Hoechst 33258 (Sigma) was added to the final wash to visualize nuclei. Infected cell foci were visualized using a Nikon Eclipse TE300 inverted epi-fluorescence microscope. EC_50_ determinations were performed as described above.

### Antiviral selectivity assays

EI-1 was serial diluted in DMSO as above and added to cells and virus during infection. Influenza virus cell protection assays were performed essentially as described [53] by infecting MDCK cells with 0.005 plaque forming units (pfu)/cell of influenza virus strain A/WS/33 (ATCC). Infected cells were incubated at 37°C for 3 days and virus-induced cytopathicity was measured using the Alamar Blue reagent (Invitrogen). BVDV cell protection assays were performed essentially as described [54] by infecting MDBK cells with 0.1 pfu/cell of BVDV strain NADL. Infected cells were incubated at 37°C for 3 days and virus-induced cytopathicity was measured using the Cell Titer-Glo reagent (Promega). HIV assays were performed as described [55] by infecting MT2 cells with 0.05 pfu/cell of the NL4-3 strain of HIV (NIH Research and Reference Reagent Program) containing the *Renilla* luciferase gene (NL-Rluc) in the Nef locus. Infected cells were incubated at 37°C for 5 days and virus replication was measured by reverse transcriptase assay using a scintillation proximity assay as described [55].

### Inhibitor time-of-addition assays

Huh-H1 cells were seeded at 5×10^3^ cells/well in 96-well plates. The following day the plates were chilled to 4°C and the media was removed and replaced with HCVpp inoculum in a volume of 50 µl. Plates were incubated at 4°C for 1.5 hr on a rocking platform. The inoculum was removed and unbound virus was removed by 2 washes with 4°C media. Fresh media was added and the plates were shifted to a 37°C incubator. 200 µg/ml porcine intestinal heparin (Sigma), 10 nM bafilomycin A1 (Sigma), 2 µg/ml anti-CD81 monoclonal antibody (BD Biosciences), or 0.125 µM EI-1 were added at specific time points during infection. At 3 days post infection, firefly luciferase activity was quantified using the Steady-Glo Reagent (Promega) according to the manufacturer's instructions. Experiments were done in triplicate and the mean % inhibition was calculated relative to control infections lacking inhibitor.

### HCVcc release assay

Huh-7.5 cells were plated in 24-well plates at 6.5×10^4^ cells/well and infected with HCVcc-1b/2a at 2 ffu/cell in the presence of EI-1 (40 or 200 nm), 8 nM NS5A inhibitor BMS-790052 [56], or DMSO control. Cells were incubated at 37°C for 5 hrs, after which the inoculum was removed and the monolayers washed 3× with phosphate buffered saline (PBS). Fresh media was added containing inhibitors or DMSO as above and the cells incubated at 37°C. Wells in which inhibitors were present continually for the duration of the infection measured the effect on entry, whereas wells that received inhibitors at 5 hrs post infection measured the effect on post-entry events. Media was removed at 2 days post infection and clarified by centrifugation at 1,000×g for 5 min. To titer the infectious HCVcc, media was serially diluted and virus quantified by indirect immunofluorescence as described above. To measure total viral particles released into the media, HCV RNA was isolated from 0.05 ml of culture supernatants using the MagMax-96 Viral RNA Isolation Kit (Ambion, Austin, TX) according to the manufacturer's protocol. Purified HCV RNA was analyzed by quantitative RT-PCR using the AgPath-ID One-Step RT-PCR Kit (Ambion) according to the manufacturer's protocol using HCV specific forward primer (5′-CGG GAG AGC CAT AGT GG-3′), reverse primer (5′-AGT ACC ACA AGG CCT TTC G-3′) and probe (5′-FAM-CTG CGG AAC CGG TGA GTA CAC-BHQ-3′) (Biosearch Technologies, Novato, CA). Samples were run on an Applied Biosystems 7900HT instrument using the 40-cycle RT-PCR protocol and the data analyzed using the SDS 2.2.2 software (Applied Biosystems, Foster City, CA). All results are the mean of triplicate assays.

### HCVcc cell-to-cell transmission assay

An assay incorporating a semisolid medium was used to access cell-to-cell spread as described [57]. HCVcc-1a/c2a was added to 1.6×10^4^ Huh-7.5 cells at 0.001 ffu/cell incubated at 37°C. At 12 hr post infection, the inoculum was removed and replaced with DMEM/2% FBS/1% Seaplaque low melting temperature agarose (Lonza, Rockland, ME) containing EI-1 (0.5 µM) or an equivalent volume of DMSO control. Cells were incubated at 37°C for 2, 3 or 4 days at which time the agarose was removed and the infected cells were detected using indirect immunofluorescence for the HCV core protein as described above. The mean number of infected cells/ffu was determined from ≥100 foci for each data point.

### HCVcc resistance selection

HCVcc-1a/2a or HCV-1b/2a was used to infect Huh-7.5 cells in media containing EI-1. Either infected cells or cell-free virus from media was serially passaged and selective pressure was progressively increased in sequential passages by raising the concentration of the inhibitor present. HCVcc replication in the presence of EI-1 was monitored by determining the spread of virus infection, using immunofluorescence, at each passage. Generally, virus stocks were prepared when HCVcc was ≥50-fold resistant relative to wild-type parental virus. The HCV genome was amplified by RT-PCR, cloned and amino acid changes that arose during inhibitor selection were identified by analysis of the DNA sequence compared to the parent and control passages in the absence of inhibitor.

### Inhibitor combination experiments

EI-1 was tested individually or in combination with NS5A inhibitor BMS-790052 [56], NS33 inhibitor BMS-605339 [58], or recombinant IFN-α-2b (Myoderm Medical Supply, Norristown, PA). Antiviral assays were performed with HCVcc-1a/2a-Rluc, and quantified as described above. Concentration-response curves were fit to the normalized responses from each inhibitor. The combination indices (CI) and Lowe's synergy were determined and analyzed as described [59]. In practice, additivity is indicated if the CI = 1.0, synergy if the CI<1.0, and antagonism if the CI>1.0. To take into account the inherent variability involved with cultured cells, the calculated 95% confidence intervals for all of the combination indices were presented. The final results were derived from 8 independent experiments for each combination.

## Results

### Identification of HCVpp inhibitors

An HCV pseudoparticle (HCVpp) infection system, utilizing a firefly luciferase reporter, was developed for high-throughput screening (HTS) of a small molecule library of >1 million compounds for inhibitors of HCV entry. In order to facilitate the screen, assay performance was improved by modifying the properties of the parental host cell line and the pseudovirus. First, we created a Huh-7B-derived cell population (Huh-H1) that overexpresses the CD81 receptor in order to improve HCVpp entry [60], resulting in an enhancement to HCVpp infection of approximately 3 fold relative to the parental Huh-7B cells (data not shown). Second, HCVpp used for the HTS contained envelope proteins E1 and E2 that were derived from genotype 1b clinical isolate 432-4 (HCVpp-1b). Infection with these pseudoparticles resulted in an approximate 3-fold increase in luciferase activity relative to HCVpp containing the prototypical 1b Con1 envelope proteins (data not shown). Third, the pseudoviruses were engineered to express a human codon optimized luciferase reporter that improved the sensitivity of the assay by increasing activity approximately 100-fold compared to wild-type firefly luciferase (data not shown).

**Figure 1:**
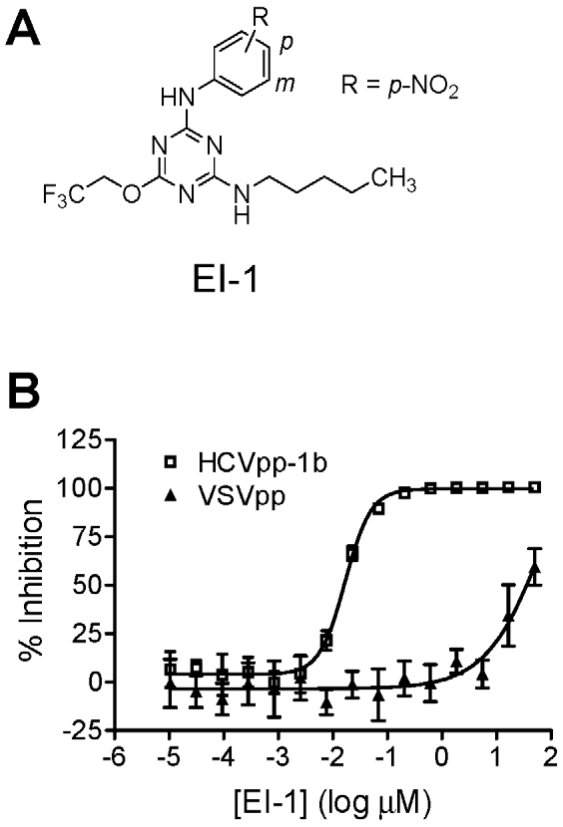
Compound structure and activity in the HCVpp assay. (A) Structure of EI-1. (B) Dose dependent inhibition of HCVpp-1b infectivity by EI-1. Huh-H1 cells were infected with HCVpp-1b or VSVpp in the presence of various concentrations of EI-1, incubated at 37°C, and luciferase activity was measured 3 days post infection. Data are presented as percent inhibition relative to control infections lacking compound. Results are expressed as mean and standard deviation from triplicate assays. EI-1 EC_50_ values are 0.016±0.001 and 33±2.1µM for HCVpp and VSVpp, respectively.

Test compounds were incubated together with HCVpp-1b and Huh-H1 cells for 2 days, at which time viral entry was quantified by luciferase activity. Compounds found to inhibit HCVpp-1b infection were then counterscreened against pseudoparticles containing the genotype 1a H77C envelope proteins (HCVpp-1a) and pseudoparticles containing the VSV glycoprotein G envelope protein (VSVpp) in order to identify HCVpp-selective inhibitors. From this process, counterscreens against other viruses, and structure-activity relationship (SAR) testing using other compounds from the collection, we identified a chemical series consisting of several structurally related compounds defined by a common triazine core. An example of one such compound, EI-1 (Fig. 1A), inhibited HCVpp-1b and VSVpp with EC_50_ values of 0.016±0.001 and 33±2.1 µM respectively, giving a VSVpp/HCVpp selectivity index of >2,000 (Fig. 1B). Other studies showed that pseudovirus with MLV envelope proteins was also not inhibited (data not shown). Because HCVpp and VSVpp differed only in their viral envelope proteins, and both pseudoparticles enter cells *via* the clathrin-mediated endocytosis pathway, the results suggested that the inhibitors targeted the HCV E1E2 proteins and not common cellular factors utilized for entry.

Synthetic chemistry efforts were used to further explore the SAR of the triazine series using EI-1 [*N* ^2^-(4-nitrophenyl)-*N* ^4^-pentyl-6-(2,2,2-trifluoroethoxy)-1,3,5-triazine-2,4-diamine] as a starting point. Our initial synthetic strategy focused on identifying an equipotent replacement for the pharmaceutically undesirable nitro group. A library of compounds was synthesized to explore the effect of substitutions at the 4′ (*para*) and 3′ (*meta*) positions of the aniline ring (Fig 1A), and these compounds were assessed for activity against HCVpp-1a and HCVpp-1b (Table 1). Removing the nitro group from EI-2 afforded a 3- to 6-fold decrease in potency in the HCVpp 1a and 1b assays, respectively. Surprisingly, the electron-donating *p*- and *m*-methoxy analogs (EI-3 and EI-9) were equipotent with the corresponding nitro group-containing analogs, while the *p*- and *m*-methyl analogs (EI-4 and EI-10) afforded HCVpp EC_50_ values within 4-fold of the corresponding nitro analogs. In contrast, introduction of the electron-withdrawing *p*-CF_3_ group (EI-5) resulted in a 15- to 45-fold decrease in 1a/1b potency in comparison with EI-1. This activity discrepancy did not extend to the *m*-CF_3_ analog (EI-11), which afforded equipotent genotype 1a/1b activity in comparison with the *m*-nitro analog (EI-8). As illustrated with EI-6 and EI-7, the introduction of *para* substituents which more closely mimicked the shape and polarity of the nitro group afforded sub-100 nM EC_50_ values in both the 1a and 1b HCVpp assays, but the HCVpp 1a activity was significantly diminished for the *meta*-substituted analogs EI-12 and EI-13. All analogs contained in Table 1 provided greater than 10-fold VSV selectivity, indicating that the activity of the compounds did not arise from cytotoxicity.

**Table 1:** Structure-activity-relationship of the EI-1 chemotype.

Sample Id	R^b^	EC_50_ (µM)^a^		
		HCVpp-1a	HCVpp-1b	VSVpp
EI-1	*p*-NO_2_	0.211	0.016	33
EI-2	H	0.721	0.115	36
EI-3	*p*-OCH_3_	0.185	0.024	33
EI-4	*p*-CH_3_	0.789	0.085	38
EI-5	*p*-CF_3_	3.219	0.933	>50
EI-6	*p*-CO_2_tBu	0.047	0.009	>50
EI-7	*p*-CONH_2_	0.042	0.007	>50
EI-8	*m*-NO_2_	0.165	0.016	11
EI-9	*m*-OCH_3_	0.095	0.054	24
EI-10	*m*-CH_3_	0.179	0.072	28
EI-11	*m*-CF_3_	0.095	0.060	11
EI-12	*m*-CO_2_tBu	1.022	0.016	14

a Mean of ≥3 independent experiments.

b R, aniline ring substituent (Fig 1A).

### EI-1 is selective for genotype 1 HCVpp

**Figure 2:**
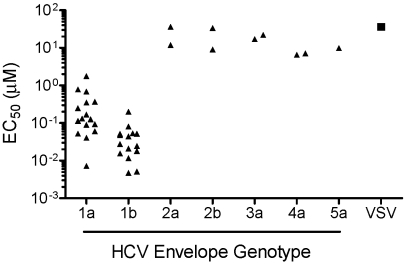
HCV genotype coverage of EI-1. Huh-H1 cells were mixed with HCVpp or VSVpp in the presence of various concentrations of EI-1. Infected cells were incubated at 37°C and luciferase activity was determined 3 days post infection. The average EC_50_ (≥2 experiments) of each of the 40 isolates representing HCV genotypes 1–5 (triangles), as well as VSVpp (square), is shown.

In order to investigate the activity of EI-1 against various HCV genotypes, we established HCVpp containing E1 and E2 proteins from different genotypic backgrounds. Serum samples from patients infected with HCV genotypes 1–5 were used as a source for cloning the E1E2 coding sequences by RT-PCR. Individual clones were then tested for the ability to support pseudovirus infectivity. From this process, a collection of HCVpp containing functional envelope proteins from each of 40 separate patient samples was obtained. The panel consists of 16 genotype 1a isolates, 15 genotype 1b isolates, 2 isolates each of genotypes 2a, 2b, 3a, and 4a, and 1 genotype 5a isolate. The potency of EI-1 was then assessed against this HCVpp panel (Fig. 2). As compared to VSVpp, EI-1 potently and selectively inhibited all 31 genotype 1 isolates with median 1a and 1b EC_50_ values of 0.134 and 0.027 µM, respectively (1a range 0.007–1.2 µM, 1b range 0.005–0.2 µM). By contrast, EI-1 inhibition of HCVpp genotypes 2–5 was minimal, with EC_50_s of 7.1 to >36 µM (equivalent to VSVpp activity, thus likely through nonselective cell cytotoxicity). Greater potency towards genotype 1 HCV was a characteristic of other related analogs tested (data not shown), providing further support to the hypothesis that the target of the compounds was likely to be a viral factor and not a general HCV entry cellular factor.

### EI-1 inhibits infection by cell culture-adapted HCV

While HCVpp are thought to enter cells in a manner analogous to authentic HCV, it was important to validate the antiviral activity of EI-1 using the fully replicating cell culture-adapted HCV (HCVcc). HCVcc luciferase reporter virus chimeras HCVcc-1a/2a-Rluc, HCVcc-1b/2a-Rluc, or HCVcc-2a/2a-Rluc expressing either genotype 1a (H77C), 1b (432-4), or 2a (J6) structural proteins, respectively, in the JFH1 background were utilized for these experiments. Huh-7.5 cells were infected in the presence of EI-1 and productive infection was determined by measuring luciferase activity at 3 days post infection. Infection by the genotype 1a/2a and 1b/2a chimeras was prevented, with EC_50_ values of 0.024 and 0.012 µM, respectively (Table 2). EI-1 was not active against genotype 2a/2a chimeric virus, consistent with the results obtained with the genotype 2a HCVpp (Fig. 2). Although EI-1 displayed similar potency against HCVcc and HCVpp expressing the genotype 1b 432–4 envelope proteins (Table 1 and Table 2), EI-1 was 8.8-fold more potent against HCVcc expressing the genotype 1a H77C envelope proteins as compared to the corresponding HCVpp. This may be related to the observation that the 1a/2a chimeric virus infection spreads more efficiently relative to the 1b/2a chimeric virus in culture (unpublished observations). The resulting additional rounds of replication can increase the apparent potency of the entry inhibitors in the HCVcc system vs.the single-cycle HCVpp assay.

**Table 2:** Activity of EI-1 in infectious virus assays.

Virus	Cell Line	EC_50_ (µM)^a^	CC_50_ (µM)^a^
HCVcc-1a/2a-Rluc	Huh-7.5	0.024	>50
HCVcc-1b/2a-Rluc	Huh-7.5	0.012	>50
HCVcc-2a/2a-Rluc	Huh-7.5	50.1	>50
BVDV	MBCK	>50	>50
Influenza virus	MDCK	>50	>50
HIV	MT2	23.4	>50

a Mean of ≥4 independent experiments.

EI-1 selectivity for HCVcc was assessed by determining the activity against a panel of viruses that enter cells by fusion with the plasma membrane (HIV) or, like HCV, undergo pH-dependent fusion with the endosomal membrane (BVDV and Influenza). In addition, compound-induced cytotoxicity in each cell line used for the infection assays was measured. No significant inhibition by EI-1 was observed against these viruses at concentrations up to the cytotoxic levels (>50 µM), which was markedly removed from the concentrations that were active against HCV (Table 2). These results support the antiviral specificity of the EI-1 HCV inhibitor.

### EI-1 inhibits an early post-attachment step of entry

**Figure 3:**
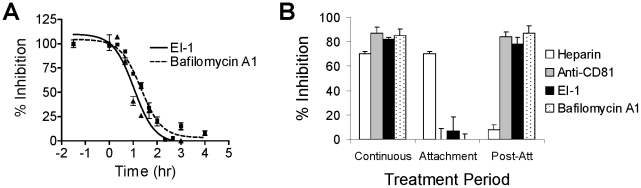
Kinetics of inhibition by HCV entry inhibitors. (A) Time course of inhibition. HCVpp (1b) was added to Huh-H1 cells at 4°C and incubated for 1.5 hr. Unadsorbed virus was removed by 2 washes with cold media, fresh media was added, and the cells were shifted to 37°C to allow synchronous infection to proceed. At the indicated time points, the media was removed and replaced with media containing 0.125 µM EI-1 (triangles and solid line) or 10 nM bafilomycin A1 (squares and dashed line) and incubated at 37° for 3 days. Inhibition was calculated as a % relative to control infections containing inhibitor throughout the experiment (100%) and those lacking inhibitor (0%). The −1.5 hr time point, in which inhibitor was present throughout the infection, represents maximum inhibition. Mean values and standard deviations were calculated from triplicate wells and the results presented are from a representative of multiple experiments. (B) Attachment and post-attachment efficacy of inhibitors. Infections were performed as described in (A). Heparin (200 µg/ml), anti-CD81 monoclonal antibody (2 µg/ml), EI-1 (0.125µM), or bafilomycin A1 (10 nM) were present in the media either continuously, during the 4°C incubation only (attachment), or during the 37°C incubation phase only (post-attachment). Inhibition was calculated as % relative to control infections lacking inhibitor. Results are expressed as mean and standard deviation from 3 independent experiments.

Although EI-1 blocked HCVcc infection, no inhibition of genotype 1a or 1b HCV replicons was observed (data not shown). To confirm that EI-1 acted at the entry stage of infection, we characterized the kinetics of compound activity using time-of-addition assays. Synchronous infection was initiated by adding HCVpp-1b to Huh-H1 cells at 4°C for 1.5 hr to allow attachment to the cell surface, presumably through HSPGs [11], [12], [13]. Unbound virus was removed and the temperature was shifted to 37°C to allow entry to proceed. EI-1 (0.125 µM) was then added at various time intervals up to 4 hrs. To define the endpoint of the entry process, bafilomycin A1, an inhibitor of endosomal acidification that prevents the final fusion step between the virus envelope and the endosomal membrane, was tested in a parallel assay. As previously reported [39], sensitivity to bafilomycin was lost at times ≥3 hrs after the 37°C temperature shift, indicating that HCVpp entry and fusion were completed within this time frame (Fig. 3A). The kinetics of EI-1 activity demonstrated that inhibition was likewise exerted within 3 hours of infection, confirming a point of action during HCVpp entry. Similar results were obtained with the HCVcc virus (data not shown).

We next examined whether EI-1 blocks the initial attachment step to HSPGs, or a downstream event in the HCV entry process. EI-1 was added together with HCVpp to cells during the 4°C attachment step only, and then removed prior to shifting to 37°C. Alternatively, EI-1 was added only following the temperature shift to measure the effect on the post-attachment events. Control inhibitors included the heparan sulfate homolog heparin, a monoclonal antibody against the CD81 receptor, and bafilomycin A1. As previously reported, heparin was only effective at preventing entry when added during the 4°C attachment step, while the CD81 monoclonal antibody and bafilomycin were only effective during the post-attachment stage (Fig 3B). EI-1 had little effect on the attachment of HCVpp to HSPGs, but exhibited >90% inhibition when added after the 37°C infection phase. Taken together, the data demonstrate that EI-1 blocks an event in HCV entry that lies temporally downstream of attachment to HSPGs, either prior to or during fusion.

### EI-1 blocks cell-to-cell spread of HCV

**Figure 4:**
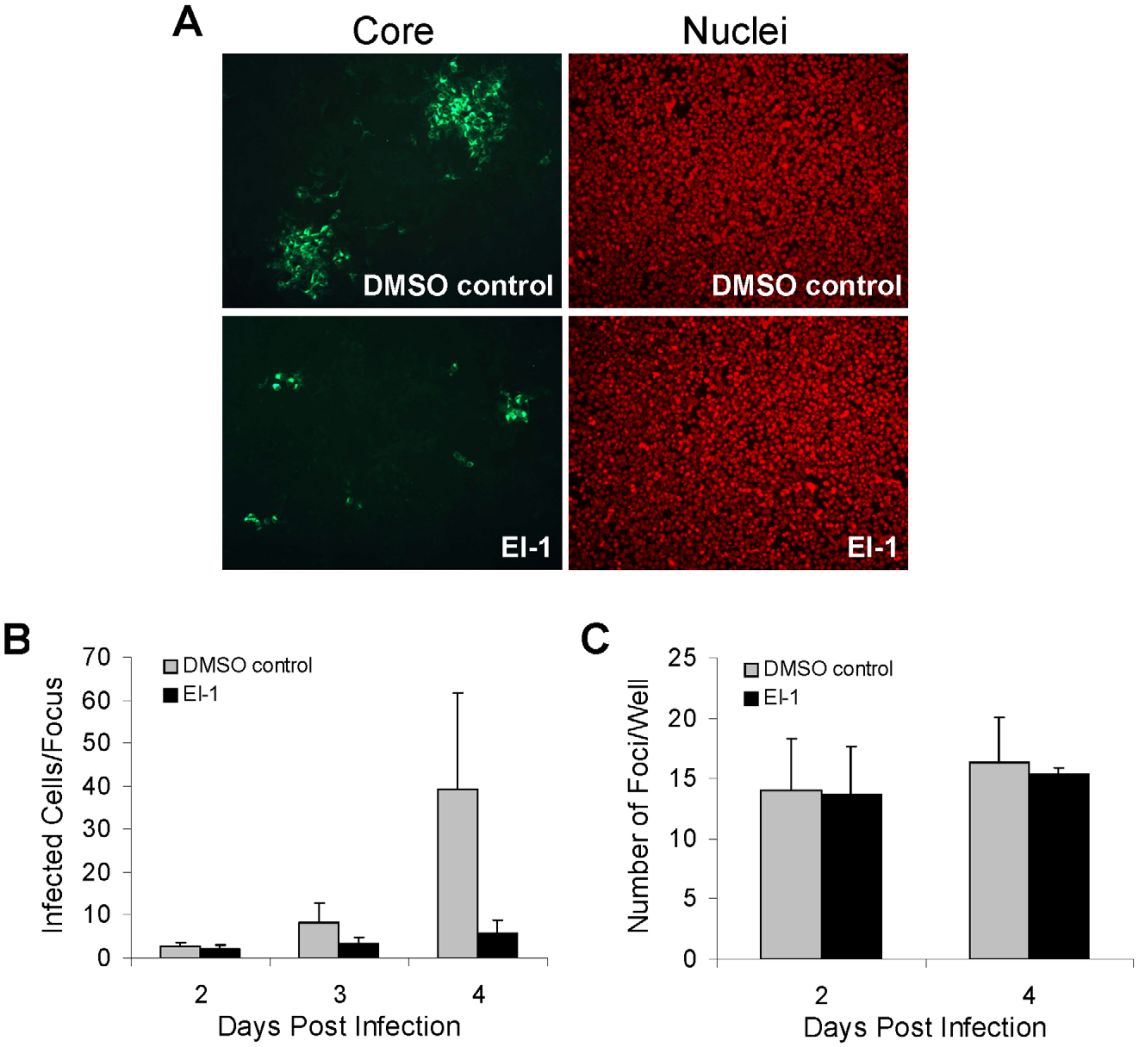
Effect of EI-1 on HCV cell-to-cell spread. (A) Huh-7.5 cells were infected with 0.001 ffu/cell HCVcc-1a/2a at 37°C. At 12 hrs post infection, the inoculum was removed and replaced with medium +1% agarose overlay containing EI-1 (0.5 µM) or DMSO and the cultures were incubated at 37°C for 2, 3 or 4 days. Infected cells were labeled by indirect immunofluorescence using an anti-HCV core monoclonal antibody (green) and nuclei were stained with Hoechst 3325 (red). Images were captured using a Nikon Eclipse TE300 inverted epi-fluorescence microscope. (B) The mean number and standard deviation of infected cells/focus was determined from visual counting of infected cells in ≥100 foci for each time point. (C) The mean number and standard deviation of foci/well was determined at 2 and 4 days post infection.

Following infection of Huh-7.5 cells with cell-free HCVcc, transmission of the virus to adjacent cells results in focal areas of spreading infection (foci). Cell-to-cell spread of HCV differs from infection with cell-free virus in that it is refractory to neutralization by HCV E2 monoclonal antibodies and occurs in a CD81-independent manner [57], [61], thus representing an alternative mode of transmission that may be important *in vivo*. We therefore conducted experiments to determine if EI-1 could block cell-to-cell spread in culture. Huh-7.5 cells were infected with the HCVcc-1a/2a chimera at a ratio of 0.001 infectious units per cell. At 12 hr post infection, the inoculum was removed and replaced with a semisolid medium/agarose overlay that has been shown to prevent the cell-free diffusion of virus but not cell-to-cell transmission [57]. Discrete foci formed in the presence of the agarose overlay lacking EI-1(Fig 4A), although they were somewhat smaller in size compared to those observed using a liquid medium (data not shown), consistent with previous observations [57]. Foci size increased over time, from an average of 2 infected cells at day 2 to an average of 40 infected cells at day 4 (Fig 4B). However, in the presence of EI-1, cell-to-cell transmission was abrogated (Fig 4A), with foci containing only an average of 6 infected cells/focus at 4 days post infection (Fig 4B). This foci size represents division of the initially-infected cell only (data not shown). In addition, the total number of foci did not increase between days 2 and 4, indicating that the agarose overlay was effective in preventing the formation of satellite foci that can result from the cell-free dissemination of virus from the primary sites of infection (Fig 4C). Overall, these results demonstrate that EI-1 is effective at preventing HCVcc infection by the cell-to-cell transmission pathway.

**Figure 5:**
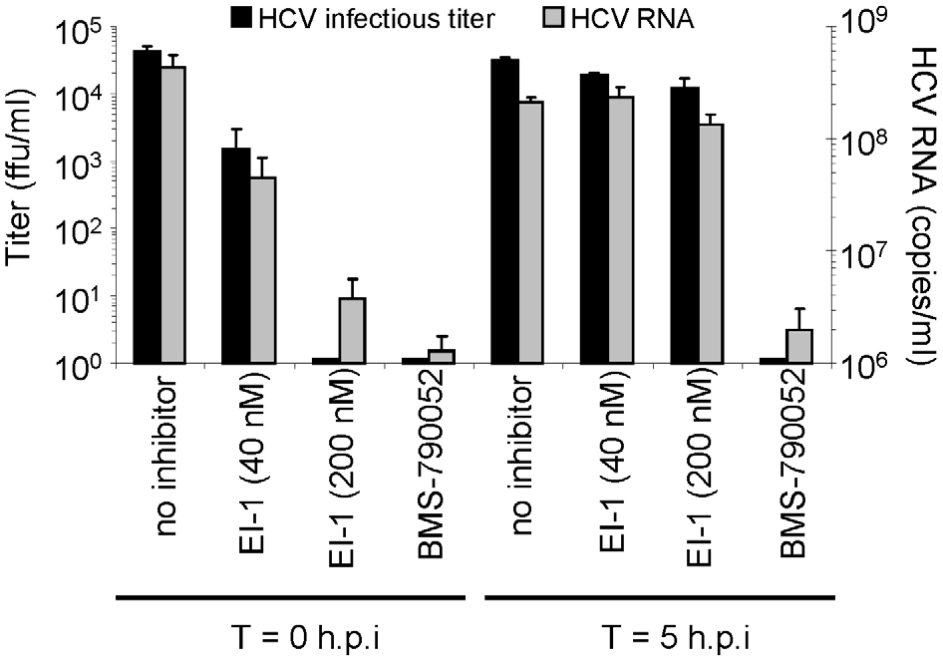
Effect of EI-1 on virus replication, assembly and release. Huh-7.5 cells were infected with HCVcc-1b/2a at 2 ffu/cell in the presence of EI-1 (40 or 200 nM), 8 nM NS5A inhibitor BMS-790052, or DMSO control. Cells were incubated at 37°C for 5 hrs, after which the inoculum was removed and the monolayers washed 3 times with phosphate buffered saline (PBS). Fresh media was added containing inhibitors or DMSO as above and the cells incubated at 37°C for 2 days. Wells in which inhibitors were present continually for the duration of the infection measured the effect on entry, whereas wells that received inhibitors at 5 hrs post infection measured the effect on post-entry events. Infectious HCVcc released into the media was quantified by indirect immunofluorescence using an anti-HCV core monoclonal antibody (ffu/ml). Total viral particles released into the media were measured by HCV-specific quantitative RT-PCR (copies/ml). Results are expressed as the mean and standard deviation of triplicate assays.

If EI-1 mediates inhibition of cell-free virus and cell-to-cell spread *via* interaction with E1 and/or E2, compound binding to the envelope protein(s) during viral replication or assembly could result in a decreased virus production or infectivity, consequently decreasing the luciferase signal and foci size. To address the potential effects of the entry inhibitor on the post-entry events of the HCV life cycle, EI-1 was added to the culture either during HCVcc infection, or 5 hours later. At two days post-infection, total virions (HCV RNA copies) and infectious virus (HCV ffu) released into the cell media were quantified. As expected, if EI-1 was present during entry, there was a dose-dependent reduction in subsequent progeny virus release (Fig 5). However, addition of EI-1 post entry did not affect the levels of virion particles or infectious virus produced. In contrast, an NS5A inhibitor BMS-790052, which targets HCV replication [56], prevented virus release when added 5 hours post infection. These results demonstrate that the EI-1 antiviral activity is confined to the pre-replication (entry) stage of infection.

### EI-1 resistance maps to the HCV E2 protein

To investigate whether E1 and/or E2 is the molecular target of EI-1, experiments were performed to determine if HCVcc resistance to the compound could be achieved by selective pressure in cell culture. HCVcc-1a/2a or HCVcc-1b/2a chimeras were propagated for several passages in the presence of increasing concentrations of EI-1. Virus was monitored for decreased susceptibility (increased EC_50_) to the compound at intervals during the selection procedure and by increased spreading of the virus through immunofluorescence detection of HCV core protein. When the HCVcc population had become ≥50-fold resistant relative to the untreated controls, and virus spreading to uninfected cells was abundant, the virus population was isolated and the genome amplified by RT-PCR. Changes in the DNA and amino acid sequence of the entire HCV polyprotein were then determined. Two independent selection experiments were conducted with independent stocks of HCVcc-1a/2a. Both EI-1 resistant virus isolates contained an amino acid substitution at residue 719 of the E2 protein (based on the H77C polyprotein numbering) in which valine changed to either phenylalanine or glycine (Table 3). E2:V719G also emerged in a third selection experiment using the HCVcc-1b/2a chimera, together with an E1:V227V/A mixture. In two of the virus populations, additional amino acid changes were also identified in non-structural proteins NS4B and NS5A.

**Table 3:** E1 and E2 amino acid changes that emerged during selection with EI-1.

Exp #	Virus	Amino Acid Substitutions			Resistance (fold/WT)^a^
		E1	E2	Other	
1	HCVcc-1a/2a	-	V719F	-	52
2	HCVcc-1a/2a	-	V719G	NS4B:T1936A, NS5A:I2345T	86
3	HCVcc-1b/2a	V227V/A	V719G	NS5A:L2249L/M	63

a Fold increase in EC_50_ compared to wild-type virus. Values represent the mean of ≥3 experiments.

To determine the role each of the amino acid changes in the EI-1 resistance phenotype, substitutions were introduced separately into wild-type HCVcc or HCVpp by site-directed mutagenesis. Substitutions that arose in NS4B and NS5A seemed unlikely to contribute to resistance of an HCV entry inhibitor. To test this hypothesis, the single E2:V719G substitution was created in the HCVcc-1a/2a chimera and the resistance level of this clone was compared to the EI-1-selected virus containing the E2:V719G+NS4B:T1936A+NS5A:I2345T (Table 3). Similar resistance levels were observed (86 vs. 91-fold/WT), suggesting the E2 mutation alone confirmed the HCVcc resistance phenotype (Table 4). Next, to confirm that the HCVcc resistance to EI-1 is mediated at the level of viral entry, each of the E1 and E2 substitutions that emerged during selection were tested in the HCVpp background. The E2:V719F and G substitutions recapitulated resistance to EI-1 in both genotype 1a and 1b HCVpp (Table 4). However, in contrast to the HCVcc results, the E2:V719G substitution in the genotype 1a background rendered the HCVpp completely resistant to EI-1 (>5,000 fold/WT). The only substitution found in the E1 envelope protein during selection with EI-1, V227A, conferred a small (∼2-fold) increase in resistance when present alone, and did not contribute significant additional resistance in combination with E2:V719G.

**Table 4:** EI-1 resistance levels of site-directed changes engineered into HCVcc or HCVpp.

Virus	Amino Acid Substitutions		Resistance (fold/WT)^a^
	E1	E2	
HCVcc-1a/2a	-	V719G	91
HCVpp-1a	-	V719F	53.5
HCVpp-1a	-	V719G	>5,000
HCVpp-1b	V227A	V719G	89.8
HCVpp-1b	V227A	-	1.9
HCVpp-1b	-	V719G	77.0
HCVpp-1a	-	V719L	17
HCVpp-1a	-	V719A	317

a Fold increase in EC_50_ compared to wild-type virus. Values represent the mean of ≥3 experiments.

**Figure 6:**
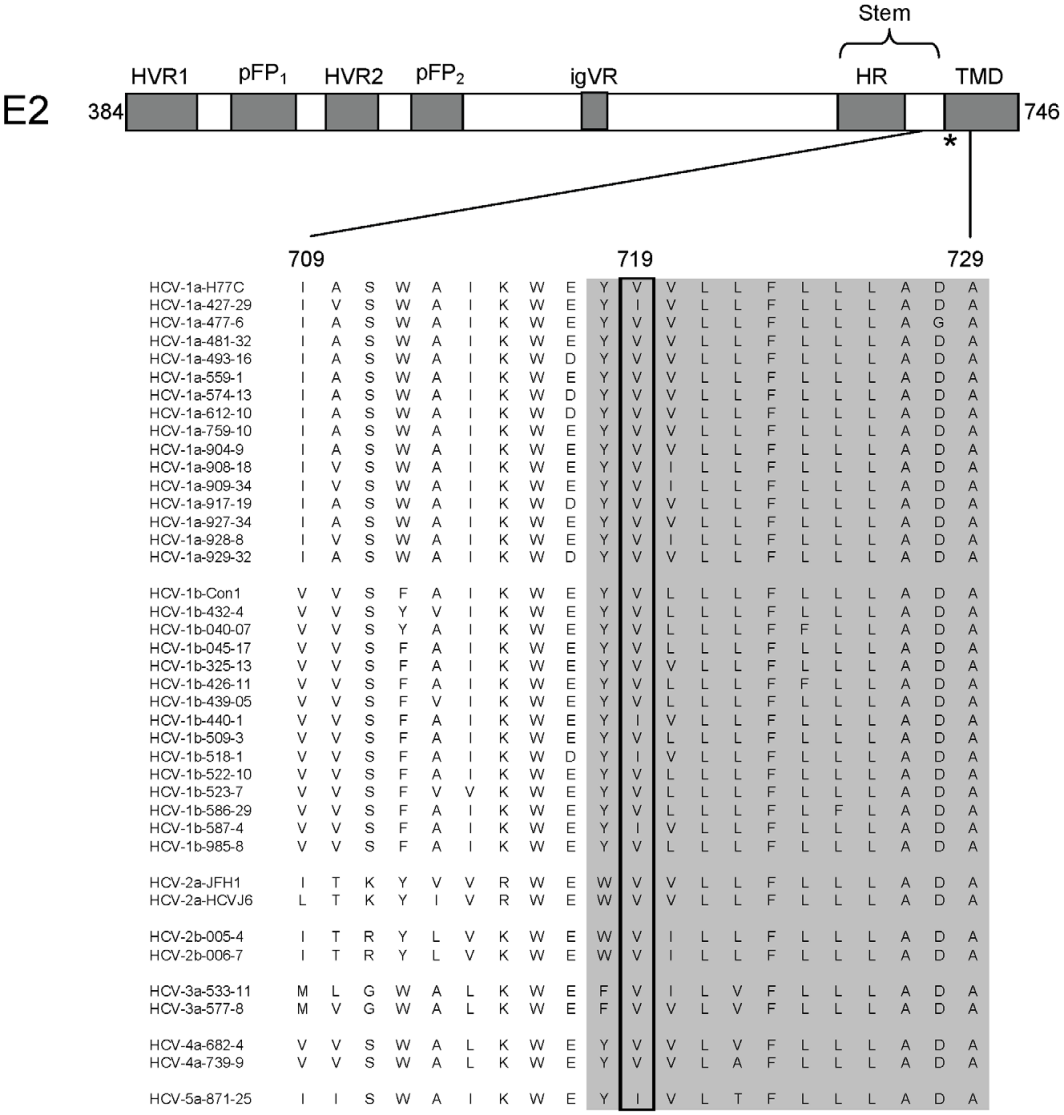
Schematic diagram of the HCV E2 protein and sequences of the region encompassing the EI-1 resistance residue. Previously defined regions of the protein are indicated by the shaded boxes. Numbers correspond to the HCV polyprotein amino acid positions in E2. HVR1, hypervariable region 1. HVR2, hypervariable region 2. pFP_1_ and pFP_2_, putative fusion peptide regions. igVR, intergenotypic variability region. HR, heptad repeat. TMD, transmembrane domain. The asterisk indicates the position of residue 719 that is involved in EI-1 resistance. The protein sequence alignment from amino acids 709–729 for each of the HCVpp genotype isolates from Figure 2 is shown. The shaded residues are part of the TMD.

Residue 719 is located within the proposed carboxy-terminal TMD of E2 near the interface with the ectodomain (Fig 6). Examination of E2 sequences containing this region in public database repositories revealed that 719V is the predominate amino acid, present in 85% of sequences. The resistance changes identified here, V719F and V719G, were not found. Other naturally occurring polymorphisms at E2:719 are isoleucine, leucine, and alanine. E2:719I is found in 14% of sequences (typically genotypes 1b, 5, and 6). This variant is represented in genotype 1a, 1b, and 5a isolates in our HCVpp genotype panel (Fig. 6), yet the genotype 1 HCVpp were fully susceptible to EI-1 (1b EC_50_ range 0.016–0.052 µM, 1a EC_50_ 0.041 µM). V:719L or A was found in only 1% of publicly available sequences. These variants were constructed by site-directed mutagenesis and found to impart moderate (V719L, 17-fold WT) to high (V719A, 317-fold WT) levels of resistance to EI-1 (Table 4). Overall, the results implicate changes to E2 residue 719 in EI-1 resistance in genotype 1 HCV. However, HCVpp panel isolates from genotypes 2–5, which are intrinsically resistant to EI-1, contain E2:719V or I, suggesting susceptibility is likely modulated by other residues or regions as well (Fig. 6).

### Entry and replication inhibitor are efficacious when combined

Due to the high rate of preexisting or emerging viral resistance, effective treatment of HCV-infected patients will likely require a combination of inhibitors targeting distinct viral or host functions. Therefore, we performed experiments using the HCVcc-1a/2a-Rluc chimera to characterize the antiviral effects of the EI-1 entry inhibitor in combination with HCV replication inhibitors targeting NS5A (BMS-790052) [56] or NS3 (BMS-605339) [58], or IFN-α. Each inhibitor was tested alone or in combination with entry inhibitor EI-1. The data from 8 replicate experiments were analyzed for departure of the results from additivity at the EC_50_ level for each combination using the Loewe model as described by Chou [59]. By this method, a combination index (CI) equal to 1 indicates additivity, and a CI<1 or >1 indicates synergy or antagonism, respectively. Combinations of EI-1 with the NS5A inhibitor or IFN-α resulted in CIs of 0.95–0.96, reflective of additivity (Table 5). The NS3 protease inhibitor combined with EI-1 gave a CI of 0.82, indicative of a moderate degree of synergy. Similar results were also obtained for all combinations at the EC_90_ level (data not shown). The absence of antagonism likely results from the mechanistically distinct targets of the entry and replication inhibitors, and indicates that entry inhibitors could provide a valuable component of combination therapy.

**Table 5:** Combination indices of EI-1 with HCV replication inhibitors.

Inhibitor Combination^a^	EC_50_ b		Loewe CI^c^	95% Confidence Interval	Result
	EI-1	2nd Inhibitor			
Entry+NS5A	0.039 µM	0.021 nM	0.95	0.90–1.00	additivity
Entry+NS3	0.029 µM	0.123 µM	0.82	0.76–0.89	moderate synergy
Entry+IFN-α	0.029 µM	1.28 IU/ml	0.96	0.90–1.01	additivity

a NS5A, BMS-790052; NS3, BMS-605339.

b Mean of 8 independent experiments.

c Loewe combination index at the combined EC_50_ level.

## Discussion

HCV entry represents an attractive target for drug discovery from a mechanistic view, with opportunities to prevent multiple virus-receptor interactions and to interfere with virus-cell membrane fusion [49]. Each of these steps, although not completely defined, is likely mediated by the HCV E1 and/or E2 envelope glycoproteins. *In vitro*, proof-of-concept for inhibiting the HCV entry process has been demonstrated using cyanovirin-N that targets the *N*-linked glycans of the viral envelope proteins and prevents E2-CD81 interaction [62], neutralizing antibodies directed against the HCV E1 and E2 proteins [63], [64], [65], [66], [67], [68], antibodies against cellular receptors CD81 [17], [18], [19], [20], [21], [22] and SR-BI [17], [18], [19], [20], [21], [22], and agents that block endosomal acidification [39], [48]. *In vivo* studies using human liver-u-PA-SCID mice have also demonstrated prophylactic efficacy of anti-CD81 antibodies [69]. In the present study, we used the HCVpp system in order to isolate the entry pathway from other HCV replication functions, and undertook a screening campaign that led to discovery of a class of small molecule HCV-specific inhibitors, exemplified by EI-1. Inhibition of entry was confirmed by using time-of-addition experiments to demonstrate that EI-1 activity is confined to the first 3 hours of infection, with inhibition occurring post-attachment and closely linked to the inhibition kinetics of the endosomal acidification inhibitor bafilomycin.

EI-1 does not inhibit entry of VSVpp, which also undergoes receptor-mediated endocytosis and pH-dependent endosomal fusion, thus making cellular factors required for internalization unlikely targets of this compound. Furthermore, although EI-1 inhibited all 31 genotype 1a and 1b isolates in our HCVpp panel, activity towards isolates with envelope genotypes 2–5 was greatly diminished. This result also argues against a cellular protein as the target for EI-1 as such an inhibitor should display similar activity across genotypes. Lastly, genotype 1 HCVcc resistance to EI-1 is conferred by a V719F/G change in the C-terminal TMD region of HCV E2, supporting the concept that EI-1 blocks HCV entry by inhibiting the function of the HCV envelope glycoproteins. It is tempting to speculate that EI-1 binds to E2 in part through an interaction with the valine or isoleucine residue 719. Alternatively, E2:V719 may represent an allosteric site, whereby changes induce a conformational alteration of E2 and/or E1 that prevent EI-1 binding. However, the E2 protein of genotypes 2–5 in our HCVpp panel also contains valine or leucine, yet these isolates are not susceptible to EI-1suggesting the determinant(s) for the intrinsic resistance of non-genotype 1 HCV may lay elsewhere. Indeed, further HCVcc resistance selection experiments suggest that changes to residues within E1 can also modulate susceptibility to other members of the EI-1 chemotype (data not shown). Ultimately, conclusive evidence for the target of EI-1 awaits biophysical experiments designed to demonstrate a direct compound-protein interaction.

It was critical to determine whether the EI-1 entry inhibitor prevented infection by HCVcc as well as HCVpp. While experimental findings obtained with the HCVpp model have generally extended to those with the HCVcc system, this is not always the case. For example, while a small molecule targeting SR-BI [70] potently inhibits HCVcc infection, it does so at a markedly reduced potency in the HCVpp system (unpublished observations). More importantly, however, it was unclear if small molecule inhibitors discovered through the HCVpp system could prove to inhibit HCVcc infection, especially since much of the HCVcc infection occurs through a cell-to-cell transmission route that is shielded from neutralizing antibodies [57] and bypasses the requirement for the CD81 receptor [61]. Since it is assumed that cell-to-cell infection is an important feature of viral pathogenesis, inhibitors that operated through both prevention of cell-free virus infection and cell-to-cell spread of virus would logically be needed for therapy. Our results demonstrate that EI-1 is potent at blocking genotype 1 HCVpp and HCVcc entry, as well as direct cell-to-cell spread of HCVcc. However, because circulating HCV in patients is highly associated with lipoprotein particles [31], [52], [71], [72], [73], [74], it will be important to determine the efficacy of EI-1 and similar HCV entry inhibitors in cell culture systems using serum-derived HCV or in human liver chimeric mouse model systems [69].

The structure of either of the HCV envelope proteins has yet to be solved. However, high-resolution structural models for the related flavivirus class II envelope glycoproteins of dengue virus, tick-borne encephalitis virus, and West Nile virus have been reported in both the pre- and post-fusion states [36], [75], [76], [77]. It is unclear how the HCV E1 and E2 proteins perform the functions of the homologous proteins in other flaviviruses. However, structural features characteristic of class II viral fusion proteins, such as a membrane proximal heptad repeat and a putative hydrophobic fusion peptide have been identified within both E1 and E2 [42], [43], [46], [78]. In addition, other laboratories have used mutational analysis to ascribe E1-E2 heterodimerization, entry, and membrane fusion functions to residues in the E2 stem and TMD [79], [80]. These results have lead to the hypothesis that flavivirus glycoproteins form intermolecular hairpin motifs projecting hydrophobic fusion peptides that facilitate the final fusion of viral envelopes with cellular membranes [81]. Further defining the target of EI-1 and elucidating the mechanism of inhibition may contribute to understanding the functional roles of the HCV envelope proteins.

A dengue virus entry inhibitor, the detergent n-octyl-β-D-glucoside (β-OG) was found to bind to a hydrophobic pocket formed in a postulated hinge region between domains I and II in the viral envelope E protein [36]. Several other inhibitors of dengue virus entry were found based on an exercise of modeling candidate compounds into this pocket [82], [83]. Modeling of domains I, II and III of the dengue E protein with HCV E1 and E2 proteins suggested that the β-OG site between domains II and III localized to the HCV E2 protein and not to E1. While β-OG did not inhibit HCV entry in our hands (data not shown), it is unclear what portion of HCV E1 or E2 may be analogous to the hinge region. In contrast to the β-OG binding site, which is within the soluble fragment of dengue virus E protein, HCV resistance to the EI-1 compound described here maps to the second amino acid of the putative TMD region of genotype 1 E2. Perhaps multiple binding sites within the HCV entry proteins exist, accessible during the numerous conformational states that may operate during receptor binding and fusion. Consistent with this concept is the finding that during our own discovery efforts, several HCV entry inhibitors with diverse structural characteristics and resistance mapping were identified (data not shown). Downstream implications of these findings are the possibility that multiple, diverse inhibitors of HCV entry could contribute to combination therapy for HCV.

Similarities between the HCV entry inhibitors described here and diverse compounds inhibiting the entry of arenaviruses into cells are intriguing. Both pseudotype [84] and infectious virus screening [85] identified broadly active arenavirus entry inhibitors. Isolation and mapping of resistant viruses, as well as chimeras between sensitive and resistant strains, mapped the target of activity to the GP2 subunit of the G envelope protein complex, specifically the interface between the C-terminal stem and TMD domains [84], [85]. These results are strikingly similar to those described in the current work in that resistance to EI-1 occurs in the same region of the genotype 1 HCV E2 envelope glycoprotein Mechanistic studies showed that these arenavirus inhibitors prevented low-pH-induced fusion by blocking reorganization between the GP2 stem with N-terminal domains of the G protein complex [86]. By analogy, perhaps the HCV entry inhibitors described here prevent pH-induced reorganization of the HCV E1E2 complex that mediates fusion [81].

The activity surrounding the search for antivirals targeting HCV is considerable. Each antiviral therapy is accompanied by a unique set of challenges for its development. Although HCV entry inhibitors could be a valuable component of therapy, their development will provide differences from those of replication inhibitor compounds, currently in clinical development. Preclinical development of entry inhibitors require infection assay formats, with pseudotyped or full-length HCV, such as those described here. While capable experimental systems have been developed, these are not as robust as many virus systems such as HIV, influenza or herpesviruses. HCV replication inhibitor assays, on the other hand, require assays with the more facile stable, transformed, replicon cell line. The inherent genetic variation in viral envelope proteins also presents a unique target for entry inhibitors. We have addressed these issues with various assay systems and an HCVpp genotype panel assembled from patient isolates, which demonstrated the genotype 1 specificity of EI-1. Furthermore, as noted above, it has been reported that the viral envelope lipoprotein content differs between cell culture HCV and virus isolated from patients. Additionally, cellular receptors and entry processes may vary from the transformed cells used here and primary hepatocytes. For these reasons, it will be important to evaluate the efficacy of small molecule HCV entry inhibitors in primary cells, using patient-derived virus, and potentially in *in vivo* model systems. Finally, current HCV inhibitor clinical studies have been limited in duration to prevent the development of resistance. The efficacy of these replication inhibitors, however, can quickly be assessed through circulating levels of HCV RNA produced by chronically-infected cells. Since entry inhibitors will prevent new infections of uninfected cells, they will have no immediate impact on the levels of circulating virus in the blood. Thus, more protracted clinical studies may be required.

Several small molecule inhibitors have been advanced to the clinic, and some have progressed after an initial high attrition rate [87]. Unfortunately, both the high replication and error rates of the viral polymerase leads to exceeding diversity of viral sequences, thus resulting in preexisting and rapidly emerging resistance [9], [10], [88]. Despite potent efficacy, it is now well understood that combinations of inhibitors, including both small molecules targeting the virus and interferon regimens acting through host targets, will be required for optimal treatment [9], [88]. By analogy to HIV, safe, potent inhibitors of multiple viral targets will be needed to prevent resistance from emerging or for optimal management of patients with resistance. We have shown the EI-1 entry inhibitor functions additively or synergistically with other HCV replication inhibitors and IFN-α in the HCVcc cell culture assay system. Entry inhibitors, such as those described here, by virtue of their distinct, relatively new targets, may provide a valuable component in the eventual optimal therapy for HCV infection.
